# Tele-ICU platform model: point of care equipment telemetry and real-time remote critical assistance

**DOI:** 10.62675/2965-2774.20260446

**Published:** 2026-06-02

**Authors:** Cleidson Cavalcante, Paulo Miranda Cavalcante, Walter Guerra, André Rodrigues, Aldenor Martins, Thais Suemi Yokoyama, Pedro Rizzi de Oliveira, Marcelo Brito Passos Amato, Carlos Roberto Ribeiro de Carvalho

**Affiliations:** 1 Universidade de São Paulo Faculdade de Medicina Hospital das Clínicas São Paulo SP Brazil Laboratory of Pulmonology LIM-09, Discipline of Pulmonology, Instituto do Coração, Hospital das Clínicas, Faculdade de Medicina, Universidade de São Paulo - São Paulo (SP), Brazil.; 2 Universidade de São Paulo Faculdade de Medicina Hospital das Clínicas São Paulo SP Brazil Division of Pulmonology, Instituto do Coração, Hospital das Clínicas, Faculdade de Medicina, Universidade de São Paulo - São Paulo (SP), Brazil.; 3 Signove Tecnologia S.A. Campina Grande PB Brazil Signove Tecnologia S.A. - Campina Grande (PB), Brazil.; 4 Universidade de São Paulo Faculdade de Medicina Hospital das Clinicas São Paulo SP Brazil Digital Health, Center for Technological Innovation, Hospital das Clinicas, Faculdade de Medicina, Universidade de São Paulo - São Paulo (SP), Brazil.

**Keywords:** Tele-ICU, Telemetry, Smart-ICU, Tele-rounds, Internet of things, Internet of Medical Things, Software, Telemedicine, Ventilators, mechanical, Clinical decision-making, Intensive care units

## Abstract

**Objective:**

To describe the requirements for modeling the tele-ICU platform, its development, the process, software, and hardware technologies employed. We also present its performance based on a proof-of-concept in a remote intensive care unit environment.

**Methods:**

This multicenter, prospective implementation study was conducted in three Level III Intensive Care Units in distinct Brazilian regions between June 2021 and December 2022. The INTEGRARE^®^, an Internet of Medical Things-based hardware/software architecture enabling agnostic integration of multiparameter monitors and mechanical ventilators, was deployed to provide continuous high-frequency telemetry, a unified analytical dashboard, and synchronous audiovisual communication for tele-round sessions. The proof-of-concept comprised continuous multimodal monitoring, collaborative tele-round discussions, and structured knowledge transfer. Nineteen months of multiparameter monitors and mechanical ventilators data were processed through a four-step data workflow (edge server, cloud storage, preprocessing, and analytical layer). All clinical, operational, and performance metrics were automatically generated by the platform.

**Results:**

The INTEGRARE^®^ enabled integration of multiparameter monitors and mechanical ventilator devices across 30 intensive care unit beds, generating over 2 billion data points with a median acquisition frequency of ~1 second and cross-device synchronization under 5 seconds. A total of 361 patients were monitored (7,235 intensive care unit-days), with a median intensive care unit stay of 14 days and 11 days on mechanical ventilators among ventilated patients. Tele-round sessions completed 484 hours, with a median of 3 hours and 39 minutes per intensive care unit per week. The platform supported real-time visualization and retrospective review of physiological curves, ventilator mechanics, laboratory results, and imaging within a unified dashboard. High adherence to tele-round routines was observed across multidisciplinary teams, who spontaneously incorporated multimodal telemetry into case discussions. Immediate bedside impact was common, including rapid identification and correction of patients’ ventilator asynchrony.

**Conclusion:**

The *Tele-UTI Conectada* model provides a technical infrastructure that supports standardization, situation awareness, and collaborative clinical decision-making by integrating real-time telemetry with synchronous audiovisual interaction across heterogeneous intensive care units. Multidisciplinary teams successfully adopted the platform and routinely used it to conduct structured case discussions.

## INTRODUCTION

Intensive care medicine is one of the most demanding fields in healthcare.^(1)^ It requires highly specialized professionals capable of interpreting a vast volume of data from multiple sources, presented in heterogeneous formats, and often lacking temporal synchrony. This complexity imposes an enormous cognitive burden on clinicians as they attempt to construct coherent clinical reasoning.^(2)^

In this context, the evolution of tele-intensive care unit (tele-ICU) systems, initially based exclusively on video communication technologies, has demonstrated improvements in supporting other intensive care units (ICUs) teams through specialized teleconsultations.^(3)^ However, significant challenges persist regarding the quality, completeness, and consistency of shared clinical information.^(3,4)^ At the same time, telemetry has long proven to be a robust solution for the continuous and structured monitoring of complementary physiological parameters in critical care, even before the consolidation of tele-ICU services.^(5,6)^

Since 2020, the coronavirus disease 2019 (COVID-19) pandemic has catalyzed research, development, and innovation initiatives involving industry, academia, and government, all aimed at mitigating its impact.^(7–9)^ As a result, based on telemetry and tele-ICU technologies, the Respiratory ICU (URe*s*) at *Instituto do Coração* of the *Hospital das Clínicas* of the *Faculdade de Medicina* of the *Universidade de São Paulo* (FMUSP) and Lifemed Company developed the *Tele-UTI Conectada* concept. This initiative resulted in the creation of technological modules capable of integrating data from bedside electromedical equipment, telemetry systems, and multimedia interactivity. The goal was to overcome limitations in diagnostic and therapeutic precision and to promote collaborative knowledge transfer between a local ICU team and a remote critical care specialist.^(10,11)^

The Respiratory ICU was the first intensive care unit to test and validate this combined telemetry and multimedia-interactivity model, applying it both to monitor its 10 beds and to facilitate case discussions among its critical care specialists. Thus, the objective of the present study was to describe the requirements for modeling the tele-ICU platform, its development, the process, software, and hardware technologies employed. We also present its performance based on a proof-of-concept in a remote ICU environment.

## METHODS

### Study design

This multicenter, retrospective study, conducted between June 2021 and December 2022, describes the implementation of a proof-of-concept *Tele-UTI Conectada* at three ICUs. The process, software, and hardware technologies designed according to the proposed model constitute the core methodological components of this study.

Accordingly, we first describe the telemetry and tele-ICU modules, later named as INTEGRARE^®^. Next, we detail how the INTEGRARE^®^ platform's intrinsic capabilities align with the *Tele-UTI Conectada* process technology model. Finally, we presented the tools and methods applied during the proof-of-concept implementation.

### Ethical considerations

No clinical records or sensitive patient information were collected, accessed, or analyzed in this study. All data were automatically generated by the INTEGRARE^®^ platform and stored without direct or indirect patient identifiers. Prior to analysis, the dataset was anonymized to ensure that individual patients could not be identified or re-identified.

### Telemetry and tele-ICU technological model

The telemetry and tele-ICU modules were developed based on the identified needs for diagnostic and therapeutic support in critical care, as well as effective knowledge transfer between remote care teams. Consequently, for the end users, the INTEGRARE^®^ platform evolved into a technological model that integrates synchronous video communication tools with simultaneous visualization of multiparameter monitor (MM) and mechanical ventilator (MV) parameters within a unified dashboard.

### INTEGRARE^®^ platform (hardware-software architecture)

**Figure 1:**
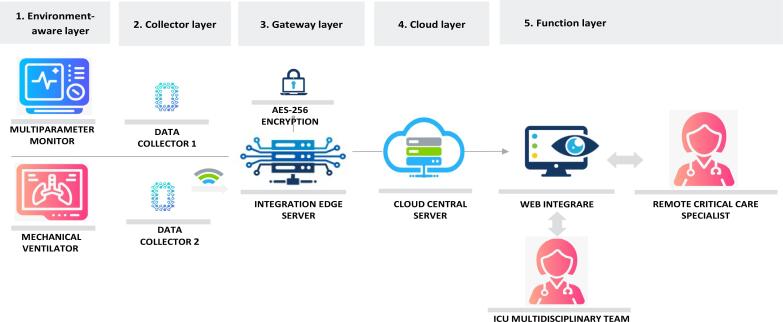
INTEGRARE platform architecture, telemetry and tele-ICU layers.

The INTEGRARE^®^ platform is based on a hardware-software architectural model that enables bidirectional communication across different critical care environments, including the bedside, the ICU multiprofessional team, and remote ICU specialists. Aligned with Internet of Medical Things (IoMT)^(12–14)^ concepts, the INTEGRARE^®^ platform is structured into five layers: 1. Environment-aware Layer; 2. Collector Layer; 3. Gateway Layer (Agnostic; Singularity; Synchronicity; Acquisition Temporality; Security); 4. Cloud Layer; and 5. Function Layer (Figure 1; see all details of the premises, five layers of architecture, and hardware-software components are described in the Supplementary Material [INTEGRARE Platform IoMT Architecture]).

ICU - intensive care unit.

**Figure 2:**
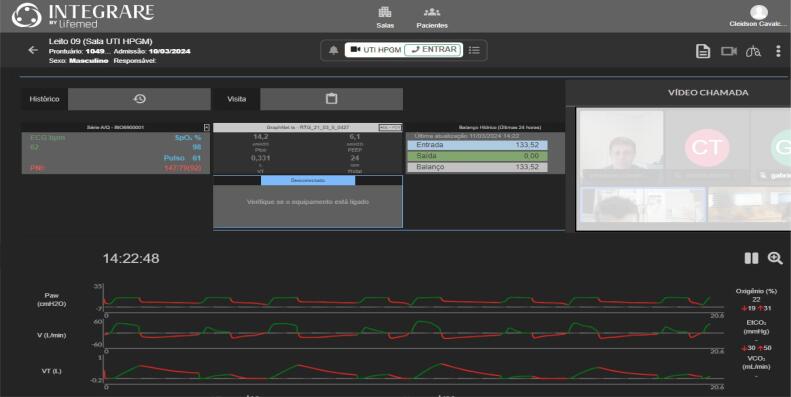
WEB INTEGRARE^®^ module panel. The number 1 represents the identification of the patient, the number of the bed, hospital admission data, and medical record ID; 2 demonstrates the hospitalization history, 3 and 4 represent the dashboard displaying the patient's clinical data during the interaction period in real time, and 5 shows the participation of the multidisciplinary intensive care unit team at a different hospital.

Inserted in the ICU environment, particularly at the bedside, the first layer (1. Environment-Aware Layer) comprises the base formed by MM and MV. These devices act as the fundamental nodes of IoMT, acquiring alarm information, numerical parameters, and physiological curves in near real time (≅1s) (Figures 1S and 2S - Supplementary Material). At the last layer of architecture, there is the Function Layer represented by the WEB INTEGRARE^®^ module (Figure 2).

**Figure 3:**
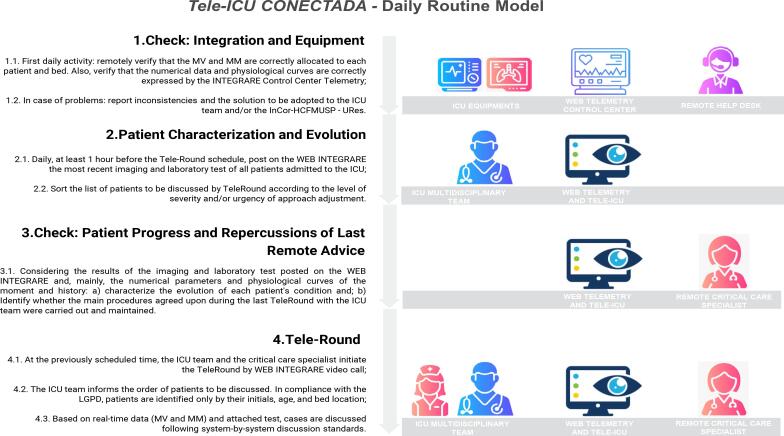
*Tele-UTI Conectada* workflow supported by the INTEGRARE^®^ platform. The figure summarizes the four steps of the tele-ICU process: (1) equipment and data integration checks; (2) patient characterization based on daily clinical, imaging, and laboratory data; (3) assessment of patient progress after the last remote advice; and (4) tele-round conducted jointly by the intensive care unit team and the remote critical care specialist.

The WEB INTEGRARE^®^ module represents the final layer of the IoMT. It consists of a unified, web-based dashboard that consolidates graphical tools for real-time visualization and interpretation of multimodal physiological data. The platform integrates waveform and numerical telemetry, laboratory results, medical imaging, and video call capabilities, enabling dynamic analysis of patient physiology within a single interface. By centralizing multiple data modalities in near real time, the module supports integrated clinical assessment during tele-ICU activities and facilitates synchronized data interpretation among multidisciplinary teams through continuous telemetry (Figure 3S - Supplementary Material).

WEB INTEGRARE^®^ consolidates detailed, multimodal physiological data into a single interface, enabling near–real-time clinical perception. Within the IoMT architecture, the Integration Edge Server enables these functionalities through core mechanisms of the singularity, data acquisition temporality, and synchronicity, ensuring semantic and temporal coherence across heterogeneous data sources (INTEGRARE Platform IoMT Architecture - Supplementary Material). Singularity addresses semantic interoperability by harmonizing equivalent clinical concepts and units of measurement generated by electromedical devices that function as independent data silos. Parameters expressed with different nomenclatures (e.g., respiratory rate as RR or Rf) are mapped to a unified data model based on international interoperability standards, ensuring consistent interpretation by healthcare professionals and automated systems, regardless of device manufacturer.^(15)^ Clinically meaningful assessment requires that physiological indicators be temporally aligned within the same observation window. To achieve this, the data acquisition temporality mechanism continuously collects data at variable rates, with an average acquisition interval of approximately 1 second, reflecting the distinct temporal requirements of hemodynamic, respiratory, and derived variables and minimizing latency in event detection. The synchronicity mechanism aligns timestamps within a predefined temporal window of ~5s, enabling parameters generated by different devices to be ordered into a coherent physiological snapshot.

### Process technology model

The *Tele-UTI Conectada* model was structured around two main components: tele-round^(16–18)^ and full multimodal monitoring. The tele-round component is defined, in accordance with Brazilian regulations,^(19)^ as the process in which a critical care specialist provides teleinterconsultation and guidance to multidisciplinary teams in other geographically distributed ICUs. Within the *Tele-UTI Conectada* model, these synchronous audiovisual interactions were instrumented by the WEB INTEGRARE^®^ module, which allows information convergence on a single dashboard, provides access to real-time physiological data, standardizes diagnostic and therapeutic routines, and consistently transfers specialized knowledge. Within the widely established hub-and-spoke concept, this model envisions the ICU's multidisciplinary team participating in tele-rounds from a dedicated physical space (meeting room) using a personal computer with web access and multimedia equipment, i.e., away from the bedside.

**Figure 4:**
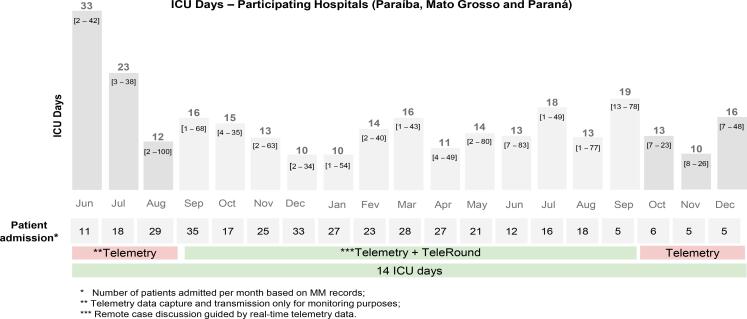
Intensive care unit days in the participating hospitals (Paraíba, Mato Grosso and Paraná).

In addition, through full multimodal monitoring, each ICU was continuously monitored 24/7 (hours/day) using real-time telemetry data from MM and MV, laboratory tests, and medical images uploaded via the WEB INTEGRARE^®^ module (Figure 4S - Supplementary Material).

This configuration enables both local ICU multidisciplinary teams and remote critical care specialists to monitor ongoing procedures and assess patient progress asynchronously at any time.

The daily operational flow of this is summarized in figure 3, which illustrates the activities performed by the multidisciplinary ICU team and the remote specialists through the INTEGRARE^®^ platform. In general, these activities included verifying system integration and equipment status, characterizing patients and updating their condition, and conducting tele-rounds based on telemetry.

ICU - intensive care unit; MM - multiparameter; MV - mechanical ventilator.

### Intensive care unit infrastructure implementation

Similar to the URes, the implementation of hardware, software, and process infrastructure was planned for the remote ICUs. Each unit received an INTEGRARE^®^ edge integration server, and the available MM and MV were configured by installing data collectors. These devices were then integrated and connected to the INTEGRARE^®^ server via the hospital's Virtual Local Area Network (VLAN), continuously transmitting telemetry data to the edge server and, consequently, to the web-based INTEGRARE^®^ platform. In addition, a multimedia kit comprising a computer, webcam, and audio system was installed in each ICU to enable local access to telemetry data and facilitate the multidisciplinary team's participation in tele-round sessions.

### Training of the intensive care unit multidisciplinary team and specialists

The training aimed to enable the multidisciplinary team to use the INTEGRARE^®^ platform for patient access and monitoring, as well as for participation in tele-rounds. Standardized remote training sessions were conducted. In accordance with the implementation and training processes previously standardized by URes, both the remote ICU multidisciplinary teams and the URes critical care specialists received 2 hours of training on the use of the WEB INTEGRARE^®^ module. For each ICU, two tele-rounds were scheduled per week, each lasting up to one hour at previously defined times. The group of critical care specialists had extensive experience in intensive care and/or pulmonology, working at level III^(20)^ facilities provided by URes.

To ensure the effectiveness of the tele-round sessions, the remote ICU multidisciplinary team was required to include at least one physician, one nurse, and one respiratory therapist.

### *Tele-UTI Conectada* proof-of-concept implementation

The Proof-of-Concept was conducted over 19 months. During the first three months, critical care specialists-URes were trained, while continuous multimodal data acquisition was initiated and maintained throughout the entire study period. After the training phase, tele-rounds sessions were launched. Supported by the INTEGRARE^®^ platform, the activities were organized into three main categories: 1- continuous telemetry monitoring; 2- collaborative support for critical case discussions (tele-round sessions), and 3- systematic knowledge transfer during the tele-rounds.

### Data collection and analysis

The study includes qualitative components based on observations during tele-rounds sessions and quantitative components based exclusively on continuous telemetry data collected by INTEGRARE^®^ from MM and VM equipment. From raw data collection to analysis, five data processing steps are required: 1. Data collected in a continuous stream over 19 months: INTEGRARE^®^ (Integration Edge Server); 2. Central storage: Cloud Service Layer; 3. Structured export from the Cloud Service Layer: The data of interest were exported based on the MIMIC-IV's structure;^(21)^ 4. Local preprocessing, grouping, interactions, and calculations: MySQL 8.0 was used to obtain a database structure in MIMIC-IV and to process the respective data; calculations were performed using the SQL language for column grouping and table interactions, with the Python 3.12.10 (Visual Code 1.107.0 - 64 bit) pandas library; 5. Data analysis: Power BI platform toolkit version 2.112 (2022) was used for the operations used in this study, focusing on mean, median, and interquartile ranges, as well as for presentation and graph generation. Data normality was assessed using the Shapiro-Wilk test. Continuous variables must be expressed as means with standard deviations or as medians with interquartile ranges, depending on data distribution.

## RESULTS

### Tele-ICU model and characterization database

The INTEGRARE^®^ server was installed in three ICUs. The available MM (LifeTouch10, LifeMed, São Paulo, Brazil) and MV equipment (Tecme-TS, Tecme, Córdoba, Argentina, and Maquet-Servo-i, Maquet, Getinge, Gothenburg, Sweden) were integrated in an agnostic way. Primarily, access to the data generated by these MMs and VMs depended on decoding the proprietary electronic communication protocol of each brand/model of equipment and, consequently, encoding it in the edge server to receive and reproduce alarms, numerical data, and physiological curves. Largely due to the circumstances caused by COVID-19, the documented protocols for the equipment used in this project were available on public websites and/or requested by the manufacturers. The integration of these devices with the INTEGRARE^®^ server enabled continuous telemetry data transmission to the edge server, making the data accessible to the respective ICUs through the WEB INTEGRARE^®^ module.

During the data collection period, the INTEGRARE^®^ database cloud was fed with more than two billion lines of parameters (MM and MV) (Table 1), resulting from the continuous transmission of electrocardiogram (ECG) wave frequency of 155 points and VM wave frequency of 50 points with a generation-reception interval of each ≅1s (Table 1S - Supplementary Material).

**Table 1:** Telemetric data acquisition period and tele-round metrics

Period of telemetric data acquisition			Period of tele-rounds			Tele-round hour/week	MM + VM parameters lines/1s	Total size of stored data	Size data/24 hours
First record: Jun/2021	Last record: Dec/2022	Total months: 19	First month: Sep/2021	Last month: Oct/2022	Total months: 13	3h10	2,363,856,240	1.6 TB	2.8 GB

MM - multiparameter monitor; MV - mechanical ventilator.

The temporal synchronization of data between different equipment modalities (MM and MV) connected to the same patient was maintained within 5 seconds. The same metrics were maintained for assisted monitoring during routine ICU and/or during tele-rounds.

In total, the INTEGRARE^®^ platform acquired MM and MV data from 30 beds in 3 ICUs. The storage space consumption for every 10 ICU beds with continuous MM and MV data was a median of 0.93 GB/24-hour [0,55 - 1,3]. Tele-rounds lasted for 13 months, totaling 484 hours and 40 minutes.

### Intensive care unit sites and populations

The ICUs were located in three distinct socioeconomic regions of Brazil: Paraíba, Mato Grosso do Sul, and Paraná (Figure 5S - Supplementary Material). All three centers were classified as Level III ICUs,^(22)^ representing the highest standard of infrastructure and service. They served as regional referral units for critical cases and primarily treated adults (> 18 years old) with a predominantly chronic clinical profile, with all admissions related to COVID-19. Over the study period, 361 patients were assisted across 7,235 ICU-days. A median of 18 patients [11 - 27] was enrolled per month, with data collected between June 2021 and December 2022.

### Tele-round and physiological detailing

From continuous MM and MV data, the INTEGRARE^®^ platform enabled a detailed understanding of the patient's physiological profile and the correspondence between therapy and the patient's condition from admission to the ICU until physical discharge.

The total data acquired in 3 ICUs had a median of 14 [12.5 - 16] ICU days/patient, equivalent to ≅2 patients/bed per month (Figure 4). The median tele-round time recorded by the INTEGRARE^®^ platform was 3 hours and 39 minutes [3 hours and 19 minutes - 3 hours 50 minutes] per ICU, per week.

ICU - intensive care unit; MM - multiparameter.

The tele-rounds routine followed system-by-system discussion standards, for each system, laboratory documents, medical images, and prescriptions attached to the WEB INTEGRARE^®^ module were accessed simultaneously with real-time physiological data visualization. In the seasonal and pandemic context, questions and events about the respiratory system were the most requested by ICU teams from critical care specialists. In turn, the hypoxemic respiratory failure data set and ICU days were interesting targets for observing the *Tele-UTI Conectada* model and the accuracy of the INTEGRARE^®^ platform data and support. 148 patients were on ventilatory support for > 12 hours in the 3 ICUs, with a median of 11 [7.25 - 11.75] days on MV (Figure 6S - Supplementary Material).

All clinical and performance data were automatically acquired by the INTEGRARE^®^ platform for analysis of the entire patient group, segmented, and within any time window. For example, using the platform's historical data tool, it was possible to retrospectively characterize the hypoxemic respiratory failure profile by combining MM and MV variables (Tables 2S and 3S - Supplementary Material). As another example, during tele-rounds sessions, when it was important to know how protective mechanical ventilation was, using INTEGRARE^®^'s waveform visualization, freezing, and measurement tools, a critical care specialist and a multidisciplinary team simultaneously evaluated and adjusted ventilation mechanics parameters in real time (Figures 7S and 8S - Supplementary Material).

## DISCUSSION

To the best of our knowledge, *Tele-UTI Conectada* and its proof of concept represent the first report of a telemetry-supported tele-round in a multicenter tele-ICU setting based on IoMT requirements, including multimodal monitoring, data heterogeneity (numerical and waveform data), real-time data synchronization, and agnostic integration of equipment within a single technological solution. These requirements enabled accurate physiological characterization while simultaneously providing a tangible, real-time perception of patient evolution during synchronous and asynchronous interactions. Although data acquisition, integration, and consumption are structurally determining factors for the success of tele-ICU initiatives,^(20)^ their full implementation over the past decade has been largely frustrating.^(23,24)^ A considerable number of tele-ICU initiatives have remained limited to remote monitoring formats based on secondary capture of vital signs via tablets or cameras attached to carts.^(23–28)^ Only a few initiatives have incorporated telemetry technologies, and even among these, varying levels of data integration are observed, such as monitoring restricted to a single equipment model and/or the absence of dynamic evaluation elements, including real-time physiological waveforms.^(2,25,26,29–33)^ Although some telemetry platforms have evolved to monitor more than one type of life-support equipment, these initiatives are generally not agnostic, remaining restricted to a single equipment manufacturer,^(27,28,33)^ and presenting limitations related to time synchronicity between devices,^(28,34)^ as well as data acquisition temporality that is insufficient to meet real-time requirements for accurate physiological waveform representation.

In addition to the structural performance of the tele-round and Telemetry services outlined in the proof-of-concept, other aspects of *Tele-UTI Conectada* potentially related to user interaction with the WEB INTEGRARE^®^ tools were observed. Discussion time proved insufficient given the ICU teams' demand for a more in-depth discussion of parameter combinations and physiological curves: tele-rounds required approximately 50% more time than initially planned. Despite this increase in duration, physicians, nurses, respiratory therapists, and medical residents across the three ICUs spontaneously adopted the tele-round routines, even in the absence of formal incentives or mandatory participation. This high level of engagement suggests that the model was perceived as valuable by frontline teams, potentially due to the availability of more detailed physiological patient information during tele-rounds and the perceived clinical support provided. On the other hand, several studies have highlighted low acceptance of tele-ICU guidance among the ICU team.^(28)^ Additionally, when there is no perceived gain by the multidisciplinary ICU team, tele-ICU services tend to fail. This contrast warrants investigation into the underlying factors that could have stimulated interest and adherence among multidisciplinary teams. One illustrative example was the identification of patient–ventilator asynchronies. Using the WEB INTEGRARE^®^ interface, the remote specialist could identify the asynchrony, freeze the waveform, and explain both its nature and the appropriate corrective action. The correction and subsequent resolution of the asynchrony could then be visualized in real time, directly benefiting the patient. This process also appeared to trigger a cascade of secondary effects, including greater transparency, credibility, and trust in the tele-ICU intervention.

When examining aspects of tele-ICU service sustainability in other studies, aspects similar to those observed in this study are identified and, to some extent, supported by the WEB INTEGRARE^®^ tools: tangible perception of the diagnostic-therapeutic dynamic; data transparency; autonomy in decision-making; mutual trust; openness to counseling; standardization; traceable results; adherence; and systematic knowledge transfer during the care process. The adoption or non-adoption of a particular therapeutic approach, within this framework, was based on explicit data and in accordance with established standards of care.^(32)^ Thus, both parties, the critical care specialist (URes) and the ICU multidisciplinary team (remote), became more comfortable with their decision-making autonomy. Consequently, the results of the adopted procedure URes are visible in real time and/or traceable through telemetric monitoring, in a cause-and-effect cycle.

Although knowledge transfer during case discussions was substantial, several challenges remained regarding the human resource profile of ICU multidisciplinary teams, including an irregular distribution across the Brazilian territory, high turnover, and unevenness in essential training, all of which represented significant limitations.^(35)^ In this context, integrating the *Tele-UTI Conectada* model with a systematic training program will be crucial to align the ICU team with updated intensive care principles and establish standardized best-practice protocols.^(36,37)^ Another limitation of this study was the absence of automated integration of infusion pump data, which precluded real-time correlation between medication infusion rates and physiological responses. This was due to the heterogeneous availability, brands, models, and age of infusion pumps during the COVID-19 pandemic, with many devices lacking electronic data extraction capabilities. Consequently, medication information was obtained from daily prescription records rather than direct device integration. Despite these limitations, tele-round and Telemetry demonstrated significant reliability in the proposed model and their respective technologies, and the conjecture URes in the use of the INTEGRARE platform demonstrated operational feasibility and reproducibility.

## CONCLUSION

The present study demonstrated that the proposed model, along with its associated processes and software technologies, fully supports the delivery of the *Tele-UTI Conectada* service and provides additional opportunities for systematic knowledge transfer and efficiency optimization in critical care. The *Tele-UTI Conectada* model also showed that integrating heterogeneous, high-resolution data streams into a unified analytical environment is an effective strategy for strengthening the structural components required for tele-ICU operations: tangible understanding through dynamic physiology analysis, standardizing and unifying the process of understanding the patient's condition, and possibly gaining perceived benefits by the ICU teams and specialists.

Despite these functional advances, the impact of *Tele-UTI Conectada* services must be assessed in greater detail. Accordingly, as the *Tele-UTI Conectada* model continues to evolve, more comprehensive investigations examining effects on key performance indicators and quality-of-care outcomes are warranted.

## Data Availability

After publication the data will be available on demand to authors.
