# Supplementary material for: Tele-ICU platform model: point of care equipment telemetry and real-time remote critical assistance
Source: Crit Care Sci. 2026 Jun 2;38:e20260446. doi: 10.62675/2965-2774.20260446 (PMC13399245; doi:10.62675/2965-2774.20260446)
Supplement: Supplementary Material [file 2965-2774-ccsci-38-e20260446-suppl01.pdf]

Critical Care Science
https://doi.org/10.62675/2965-2774.20260446

SUPPLEMENTARY MATERIAL

Tele-ICU platform model: point of care equipment telemetry and real-time remote critical assistance

Cleidson Cavalcante[1], Paulo Miranda Cavalcante Neto[2], Walter Guerra[3], André Rodrigues[3], Aldenor Martins[3], Thais Suemi Yokoyama[4], Pedro Rizzi de Oliveira[4], Marcelo Brito Passos Amato[2], Carlos Roberto Ribeiro de Carvalho[2]

INTEGRARE PLATFORM IOMT ARCHITECTURE

The ICU bedside environment forms the first Internet of Medical Things (IoMT) layer, comprising multiparameter monitors (MM) and mechanical ventilators (MV) that function as basic nodes, acquiring alarms, numerical data, and physiological waveforms in near-real time. To enable a systemic understanding of patient physiology and its response to disease and therapy, the INTEGRARE® platform was developed using a multimodal architecture that centralizes data from diverse monitoring and life-support devices into a single electronic environment. This architecture is structured into five IoMT layers: (A) Environment-Aware, (B) Collector, (C) Gateway – responsible for integration, agnostic processing, data singularity, synchronicity, temporal acquisition, and security, (D) Cloud, and (E) Function:

A) Environment-aware layer - the core of this first layer consists of a series of network equipment data collectors and communication protocols. It is recommended that the integration of bedside equipment for telemetry and tele-ICU be agnostic, that is, independent of the brand or model of the devices used. The diversity of equipment installed in intensive care units (ICUs) is a constant challenge, as different manufacturers use proprietary formats and syntaxes; as a result, they are not standardized or interoperable with open electronic systems. Given the reality of countries like Brazil, ensuring interoperability regardless of manufacturer requires adopting approaches based on international communication standards, such as Health Level 7 (HL7), combined with technological modules that adapt to the available equipment's communication protocols. Based on these premises, in INTEGRARE® there are three node types: Data Collector, Integration Edge Server, and Cloud.

B) Data collector - for medical devices that can't communicate using TCP/IP, a dedicated piece of equipment called Lifebridge was developed. This device can physically connect to the medical device via RS-232 or another standard and send data to the Integration Edge Server over wi-fi or cabled connection.

C) Integration Edge Server - this is the central module of the INTEGRARE® platform, organized into five groups of mechanisms in line with requirements for full data integration:

C.1) Agnostic - it involves a mechanism for decoding the proprietary data formats and syntax of each brand/model of equipment, as well as the implementation capable of converging and standardizing the data collected from the equipment, where each medical device represents a data silo with a unique data model.

C.2) Concept singularity - devices that perform the same function may have heterogeneous parameters and terminology. This mechanism imports the widest possible range of data from each electromedical equipment and aligns different forms of expression of the same concept with a single form of identification.

C.3) Synchronicity - temporal synchronization between data from multiple electromedical equipment (MM and MV) is essential to ensure the representativeness of the patient's physiological moment. Therefore, this mechanism implements timestamp alignment in a temporal window of up to 5 seconds so that the different parameters and equipment are ordered in the same time interval, mirroring what occurred in the patient's physiology (Table 1S).

This is an open access article under the CC BY license (https://creativecommons.org/licenses/by/4.0/).

C.4) Data acquisition temporality - ideally, a telemetry platform should operate at sampling rates appropriate to the type of data collected - for example, hemodynamic and respiratory variables require different temporal resolutions to be clinically useful. Latency in data acquisition can compromise the care team's ability to detect relevant physiological events. To this end, these mechanisms continuously collect data from the equipment (MM and MV) at an average rate of 1 second.

C.5) Data security - to ensure data security and compliance with the Brazilian General Data Protection Law (LGPD) and other relevant regulations, INTEGRARE® adheres to established industry best practices in data handling and cybersecurity. All data transmissions are conducted over secure communication channels, and stored data is encrypted using AES-256. Access to data is restricted to authenticated users and systems with appropriate authorization levels. Both the local edge server and the cloud server are protected by firewalls configured according to the principle of least privilege. Additionally, independent third-party firms regularly conduct security audits to identify and mitigate potential vulnerabilities.

D) Cloud service layer - all telemetry data aggregated by Integration's edge server is transmitted to the cloud services layer, which provides unified data storage and analysis. Cloud services expand the capacity for distributed geographic access to the web while still maintaining data security levels.

E) Function layer - at the top, there is the last layer, where data is accessed for consumption by two main services: (1) simultaneous and synchronous telemetry center for MM and VM data, for monitoring by the multidisciplinary team (doctors, nurses, and physical therapists) in the ICU (Figures 1S, 2S) or remote; (2) simultaneous and synchronous web access (WEB INTEGRARE® module) to Multimedia Device (MD), MM and VM data, for the tele-ICU Web service (Figures 3S, 4S).

The WEB INTEGRARE® module represents the final IoMT layer, consolidating graphical tools in a single dashboard that enable real-time visualization and interpretation of multimodal physiological data. By integrating waveform and numerical information, the platform supports dynamic analysis of patient physiology and strengthens collaborative clinical decision-making. Designed to enhance users' analytical capacity and promote evidence-based assessment, the module fosters transparency during tele-ICU discussions, builds trust among multidisciplinary teams, and serves as a practical mechanism for knowledge exchange through continuous telemetry (Figure 3S).

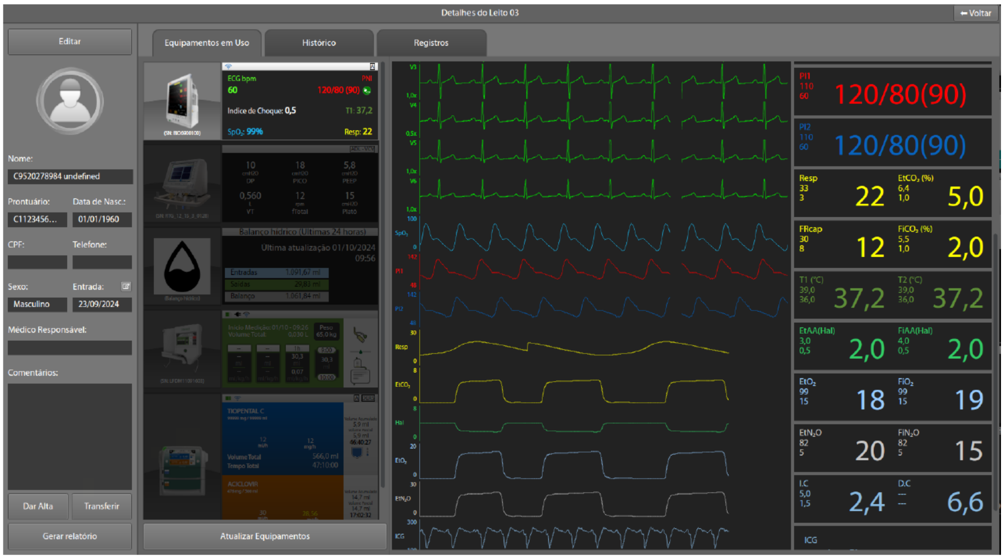

Figure 1S - INTEGRARE® local gateway layer - multiparameter monitors alarms, parameters, and physiologic waves.

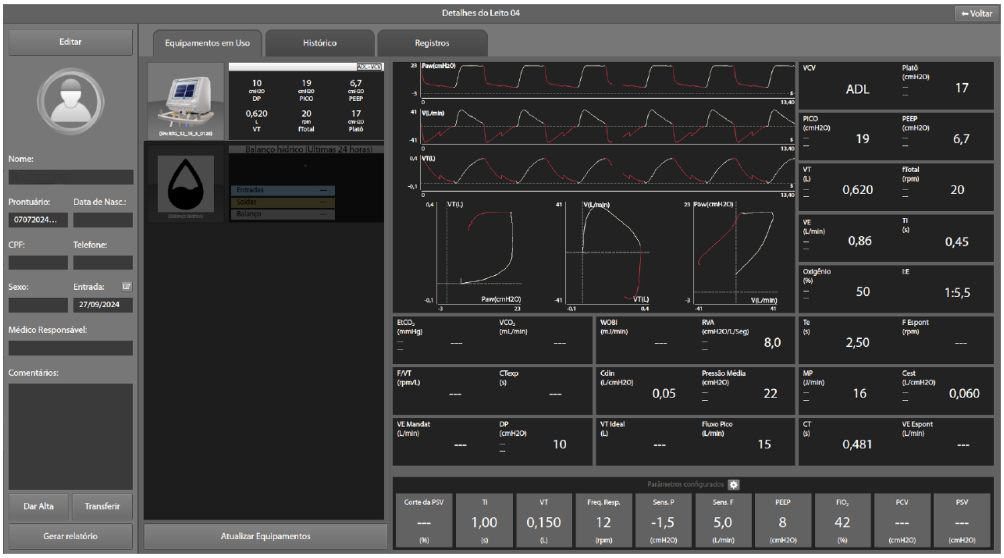

Figure 2S - INTEGRARE® local gateway layer - mechanical ventilator alarms, parameters, and ventilatory waves.

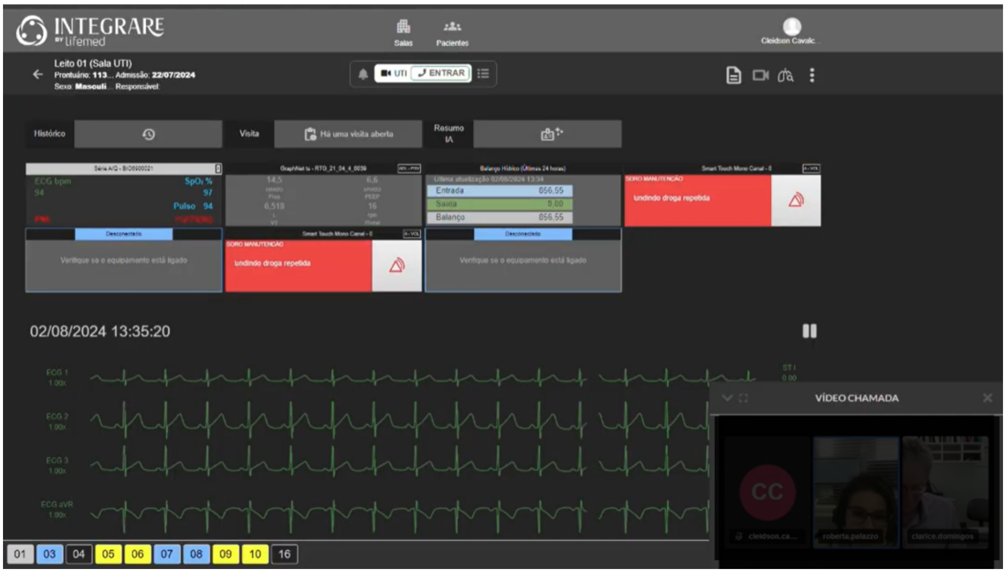

Figure 3S - WEB INTEGRARE® module - video call, multiparameter monitors and mechanical ventilator alarms, parameters, and ventilatory waves.

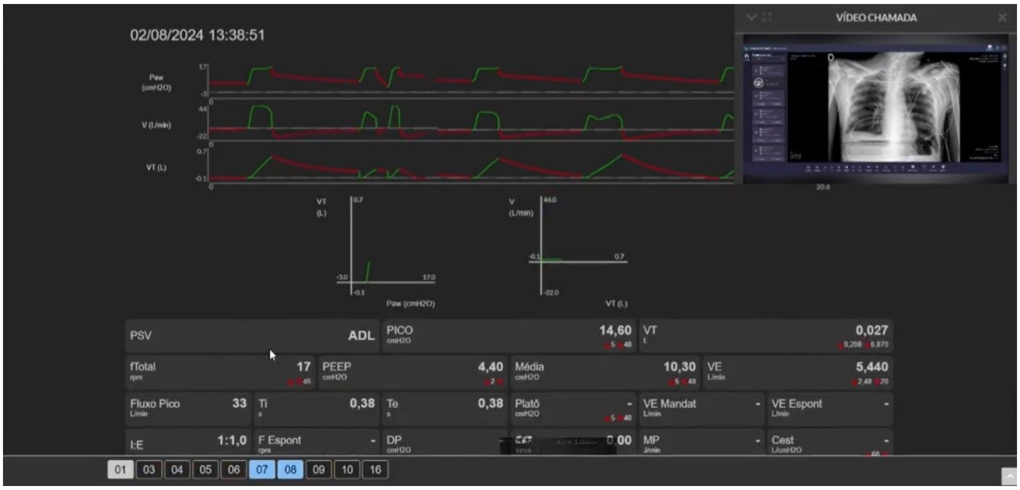

Figure 4S - INTEGRARE® module - real-time telemetry data from mechanical ventilation, laboratory tests, medical images, and video call.

TIMESTAMP LINES OF MULTIPARAMETER MONITOR/1S (“)

Table 1S - Timestamp sampling

Timestamp	Blood pressure	Electrocardiogram	Respiratory	Temperature
1725993298000	{“diastolic”: 80, “mean”: 90, “systolic”: 120}	{“heartRate”: 60, “st1”: 0, “st2”: 0, “st3”: 0, “stAvf”: 0, “stAvl”: 0, “stAvr”: 0, “stV”: 0, “stV1”: 0, “stV2”: 0, “stV3”: 0, “stV5”: 0, “stV6”: 0}	{“resp”: 22}	{“temp1”: 37.2, “temp2”: 37.2}
1725993299000	{“diastolic”: 80, “mean”: 90, “systolic”: 120}	{“heartRate”: 60, “st1”: 0, “st2”: 0, “st3”: 0, “stAvf”: 0, “stAvl”: 0, “stAvr”: 0, “stV”: 0, “stV1”: 0, “stV2”: 0, “stV3”: 0, “stV5”: 0, “stV6”: 0}	{“resp”: 22}	{“temp1”: 37.2, “temp2”: 37.2}
1725993300000	{“diastolic”: 80, “mean”: 90, “systolic”: 120}	{“heartRate”: 60, “st1”: 0, “st2”: 0, “st3”: 0, “stAvf”: 0, “stAvl”: 0, “stAvr”: 0, “stV”: 0, “stV1”: 0, “stV2”: 0, “stV3”: 0, “stV5”: 0, “stV6”: 0}	{“resp”: 22}	{“temp1”: 37.2, “temp2”: 37.2}
1725993301000	{“diastolic”: 80, “mean”: 90, “systolic”: 120}	{“heartRate”: 60, “st1”: 0, “st2”: 0, “st3”: 0, “stAvf”: 0, “stAvl”: 0, “stAvr”: 0, “stV”: 0, “stV1”: 0, “stV2”: 0, “stV3”: 0, “stV5”: 0, “stV6”: 0}	{“resp”: 22}	{“temp1”: 37.2, “temp2”: 37.2}
1725993302000	{“diastolic”: 80, “mean”: 90, “systolic”: 120}	{“heartRate”: 60, “st1”: 0, “st2”: 0, “st3”: 0, “stAvf”: 0, “stAvl”: 0, “stAvr”: 0, “stV”: 0, “stV1”: 0, “stV2”: 0, “stV3”: 0, “stV5”: 0, “stV6”: 0}	{“resp”: 22}	{“temp1”: 37.2, “temp2”: 37.2}
1725993303000	{“diastolic”: 80, “mean”: 90, “systolic”: 120}	{“heartRate”: 60, “st1”: 0, “st2”: 0, “st3”: 0, “stAvf”: 0, “stAvl”: 0, “stAvr”: 0, “stV”: 0, “stV1”: 0, “stV2”: 0, “stV3”: 0, “stV5”: 0, “stV6”: 0}	{“resp”: 22}	{“temp1”: 37.2, “temp2”: 37.2}
1725993304000	{“diastolic”: 80, “mean”: 90, “systolic”: 120}	{“heartRate”: 60, “st1”: 0, “st2”: 0, “st3”: 0, “stAvf”: 0, “stAvl”: 0, “stAvr”: 0, “stV”: 0, “stV1”: 0, “stV2”: 0, “stV3”: 0, “stV5”: 0, “stV6”: 0}	{“resp”: 22}	{“temp1”: 37.2, “temp2”: 37.2}
1725993305000	{“diastolic”: 80, “mean”: 90, “systolic”: 120}	{“heartRate”: 60, “st1”: 0, “st2”: 0, “st3”: 0, “stAvf”: 0, “stAvl”: 0, “stAvr”: 0, “stV”: 0, “stV1”: 0, “stV2”: 0, “stV3”: 0, “stV5”: 0, “stV6”: 0}	{“resp”: 22}	{“temp1”: 37.2, “temp2”: 37.2}
1725993306000	{“diastolic”: 80, “mean”: 90, “systolic”: 120}	{“heartRate”: 60, “st1”: 0, “st2”: 0, “st3”: 0, “stAvf”: 0, “stAvl”: 0, “stAvr”: 0, “stV”: 0, “stV1”: 0, “stV2”: 0, “stV3”: 0, “stV5”: 0, “stV6”: 0}	{“resp”: 22}	{“temp1”: 37.2, “temp2”: 37.2}
1725993307000	{“diastolic”: 80, “mean”: 90, “systolic”: 120}	{“heartRate”: 60, “st1”: 0, “st2”: 0, “st3”: 0, “stAvf”: 0, “stAvl”: 0, “stAvr”: 0, “stV”: 0, “stV1”: 0, “stV2”: 0, “stV3”: 0, “stV5”: 0, “stV6”: 0}	{“resp”: 22}	{“temp1”: 37.2, “temp2”: 37.2}
1725993308000	{“diastolic”: 80, “mean”: 90, “systolic”: 120}	{“heartRate”: 60, “st1”: 0, “st2”: 0, “st3”: 0, “stAvf”: 0, “stAvl”: 0, “stAvr”: 0, “stV”: 0, “stV1”: 0, “stV2”: 0, “stV3”: 0, “stV5”: 0, “stV6”: 0}	{“resp”: 22}	{“temp1”: 37.2, “temp2”: 37.2}
1725993309000	{“diastolic”: 80, “mean”: 90, “systolic”: 120}	{“heartRate”: 60, “st1”: 0, “st2”: 0, “st3”: 0, “stAvf”: 0, “stAvl”: 0, “stAvr”: 0, “stV”: 0, “stV1”: 0, “stV2”: 0, “stV3”: 0, “stV5”: 0, “stV6”: 0}	{“resp”: 22}	{“temp1”: 37.2, “temp2”: 37.2}
1725993310000	{“diastolic”: 80, “mean”: 90, “systolic”: 120}	{“heartRate”: 60, “st1”: 0, “st2”: 0, “st3”: 0, “stAvf”: 0, “stAvl”: 0, “stAvr”: 0, “stV”: 0, “stV1”: 0, “stV2”: 0, “stV3”: 0, “stV5”: 0, “stV6”: 0}	{“resp”: 22}	{“temp1”: 37.2, “temp2”: 37.2}
1725993311000	{“diastolic”: 80, “mean”: 90, “systolic”: 120}	{“heartRate”: 60, “st1”: 0, “st2”: 0, “st3”: 0, “stAvf”: 0, “stAvl”: 0, “stAvr”: 0, “stV”: 0, “stV1”: 0, “stV2”: 0, “stV3”: 0, “stV5”: 0, “stV6”: 0}	{“resp”: 22}	{“temp1”: 37.2, “temp2”: 37.2}
1725993312000	{“diastolic”: 80, “mean”: 90, “systolic”: 120}	{“heartRate”: 60, “st1”: 0, “st2”: 0, “st3”: 0, “stAvf”: 0, “stAvl”: 0, “stAvr”: 0, “stV”: 0, “stV1”: 0, “stV2”: 0, “stV3”: 0, “stV5”: 0, “stV6”: 0}	{“resp”: 22}	{“temp1”: 37.2, “temp2”: 37.2}
1725993313000	{“diastolic”: 80, “mean”: 90, “systolic”: 120}	{“heartRate”: 60, “st1”: 0, “st2”: 0, “st3”: 0, “stAvf”: 0, “stAvl”: 0, “stAvr”: 0, “stV”: 0, “stV1”: 0, “stV2”: 0, “stV3”: 0, “stV5”: 0, “stV6”: 0}	{“resp”: 22}	{“temp1”: 37.2, “temp2”: 37.2}
1725993314000	{“diastolic”: 80, “mean”: 90, “systolic”: 120}	{“heartRate”: 60, “st1”: 0, “st2”: 0, “st3”: 0, “stAvf”: 0, “stAvl”: 0, “stAvr”: 0, “stV”: 0, “stV1”: 0, “stV2”: 0, “stV3”: 0, “stV5”: 0, “stV6”: 0}	{“resp”: 22}	{“temp1”: 37.2, “temp2”: 37.2}
1725993315000	{“diastolic”: 80, “mean”: 90, “systolic”: 120}	{“heartRate”: 60, “st1”: 0, “st2”: 0, “st3”: 0, “stAvf”: 0, “stAvl”: 0, “stAvr”: 0, “stV”: 0, “stV1”: 0, “stV2”: 0, “stV3”: 0, “stV5”: 0, “stV6”: 0}	{“resp”: 22}	{“temp1”: 37.2, “temp2”: 37.2}
1725993316000	{“diastolic”: 80, “mean”: 90, “systolic”: 120}	{“heartRate”: 60, “st1”: 0, “st2”: 0, “st3”: 0, “stAvf”: 0, “stAvl”: 0, “stAvr”: 0, “stV”: 0, “stV1”: 0, “stV2”: 0, “stV3”: 0, “stV5”: 0, “stV6”: 0}	{“resp”: 22}	{“temp1”: 37.2, “temp2”: 37.2}
1725993317000	{“diastolic”: 80, “mean”: 90, “systolic”: 120}	{“heartRate”: 60, “st1”: 0, “st2”: 0, “st3”: 0, “stAvf”: 0, “stAvl”: 0, “stAvr”: 0, “stV”: 0, “stV1”: 0, “stV2”: 0, “stV3”: 0, “stV5”: 0, “stV6”: 0}	{“resp”: 22}	{“temp1”: 37.2, “temp2”: 37.2}
1725993318000	{“diastolic”: 80, “mean”: 90, “systolic”: 120}	{“heartRate”: 60, “st1”: 0, “st2”: 0, “st3”: 0, “stAvf”: 0, “stAvl”: 0, “stAvr”: 0, “stV”: 0, “stV1”: 0, “stV2”: 0, “stV3”: 0, “stV5”: 0, “stV6”: 0}	{“resp”: 22}	{“temp1”: 37.2, “temp2”: 37.2}
1725993319000	{“diastolic”: 80, “mean”: 90, “systolic”: 120}	{“heartRate”: 60, “st1”: 0, “st2”: 0, “st3”: 0, “stAvf”: 0, “stAvl”: 0, “stAvr”: 0, “stV”: 0, “stV1”: 0, “stV2”: 0, “stV3”: 0, “stV5”: 0, “stV6”: 0}	{“resp”: 22}	{“temp1”: 37.2, “temp2”: 37.2}
1725993320000	{“diastolic”: 80, “mean”: 90, “systolic”: 120}	{“heartRate”: 60, “st1”: 0, “st2”: 0, “st3”: 0, “stAvf”: 0, “stAvl”: 0, “stAvr”: 0, “stV”: 0, “stV1”: 0, “stV2”: 0, “stV3”: 0, “stV5”: 0, “stV6”: 0}	{“resp”: 22}	{“temp1”: 37.2, “temp2”: 37.2}
1725993321000	{“diastolic”: 80, “mean”: 90, “systolic”: 120}	{“heartRate”: 60, “st1”: 0, “st2”: 0, “st3”: 0, “stAvf”: 0, “stAvl”: 0, “stAvr”: 0, “stV”: 0, “stV1”: 0, “stV2”: 0, “stV3”: 0, “stV5”: 0, “stV6”: 0}	{“resp”: 22}	{“temp1”: 37.2, “temp2”: 37.2}
1725993322000	{“diastolic”: 80, “mean”: 90, “systolic”: 120}	{“heartRate”: 60, “st1”: 0, “st2”: 0, “st3”: 0, “stAvf”: 0, “stAvl”: 0, “stAvr”: 0, “stV”: 0, “stV1”: 0, “stV2”: 0, “stV3”: 0, “stV5”: 0, “stV6”: 0}	{“resp”: 22}	{“temp1”: 37.2, “temp2”: 37.2}

Continue...

...continuation

1725993323000	{"diastolic": 80, "mean": 90, "systolic": 120}	{"heartRate": 60, "st1": 0, "st2": 0, "st3": 0, "stAvf": 0, "stAvl": 0, "stAvr": 0, "stV": 0, "stV1": 0, "stV2": 0, "stV3": 0, "stV5": 0, "stV6": 0}	{"resp": 22}	{"temp1": 37.2, "temp2": 37.2}
1725993324000	{"diastolic": 80, "mean": 90, "systolic": 120}	{"heartRate": 60, "st1": 0, "st2": 0, "st3": 0, "stAvf": 0, "stAvl": 0, "stAvr": 0, "stV": 0, "stV1": 0, "stV2": 0, "stV3": 0, "stV5": 0, "stV6": 0}	{"resp": 22}	{"temp1": 37.2, "temp2": 37.2}
1725993325000	{"diastolic": 80, "mean": 90, "systolic": 120}	{"heartRate": 60, "st1": 0, "st2": 0, "st3": 0, "stAvf": 0, "stAvl": 0, "stAvr": 0, "stV": 0, "stV1": 0, "stV2": 0, "stV3": 0, "stV5": 0, "stV6": 0}	{"resp": 22}	{"temp1": 37.2, "temp2": 37.2}
1725993326000	{"diastolic": 80, "mean": 90, "systolic": 120}	{"heartRate": 60, "st1": 0, "st2": 0, "st3": 0, "stAvf": 0, "stAvl": 0, "stAvr": 0, "stV": 0, "stV1": 0, "stV2": 0, "stV3": 0, "stV5": 0, "stV6": 0}	{"resp": 22}	{"temp1": 37.2, "temp2": 37.2}
1725993327000	{"diastolic": 80, "mean": 90, "systolic": 120}	{"heartRate": 60, "st1": 0, "st2": 0, "st3": 0, "stAvf": 0, "stAvl": 0, "stAvr": 0, "stV": 0, "stV1": 0, "stV2": 0, "stV3": 0, "stV5": 0, "stV6": 0}	{"resp": 22}	{"temp1": 37.2, "temp2": 37.2}

Timestamp lines of multiparameter monitor / 1 second ("): blood pressure; electrocardiogram; respiratory; temperature.

INTENSIVE CARE UNIT SITES

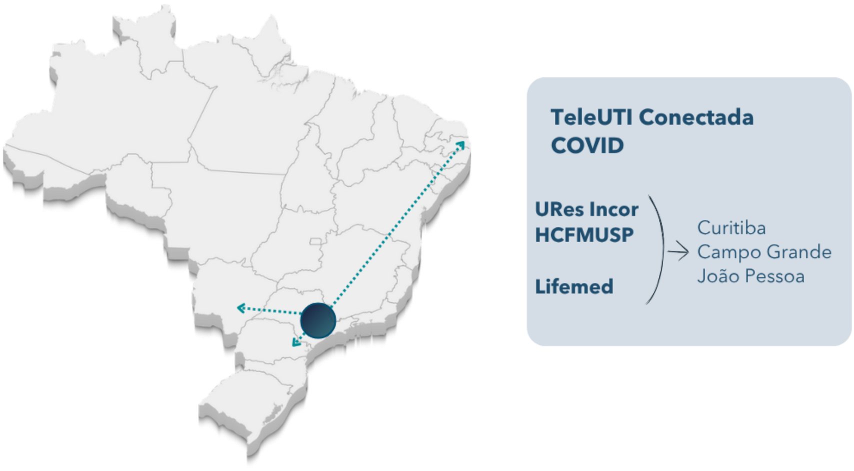

HCFMUSP - *Hospital das Clínicas* of the *Faculdade de Medicina* of the *Universidade de São Paulo*.

Figure 5S - The intensive care units were in three distinct socioeconomic regions of Brazil: Paraíba, Mato Grosso do Sul, and Paraná.

TELE-ROUND AND PHYSIOLOGICAL DETAILING

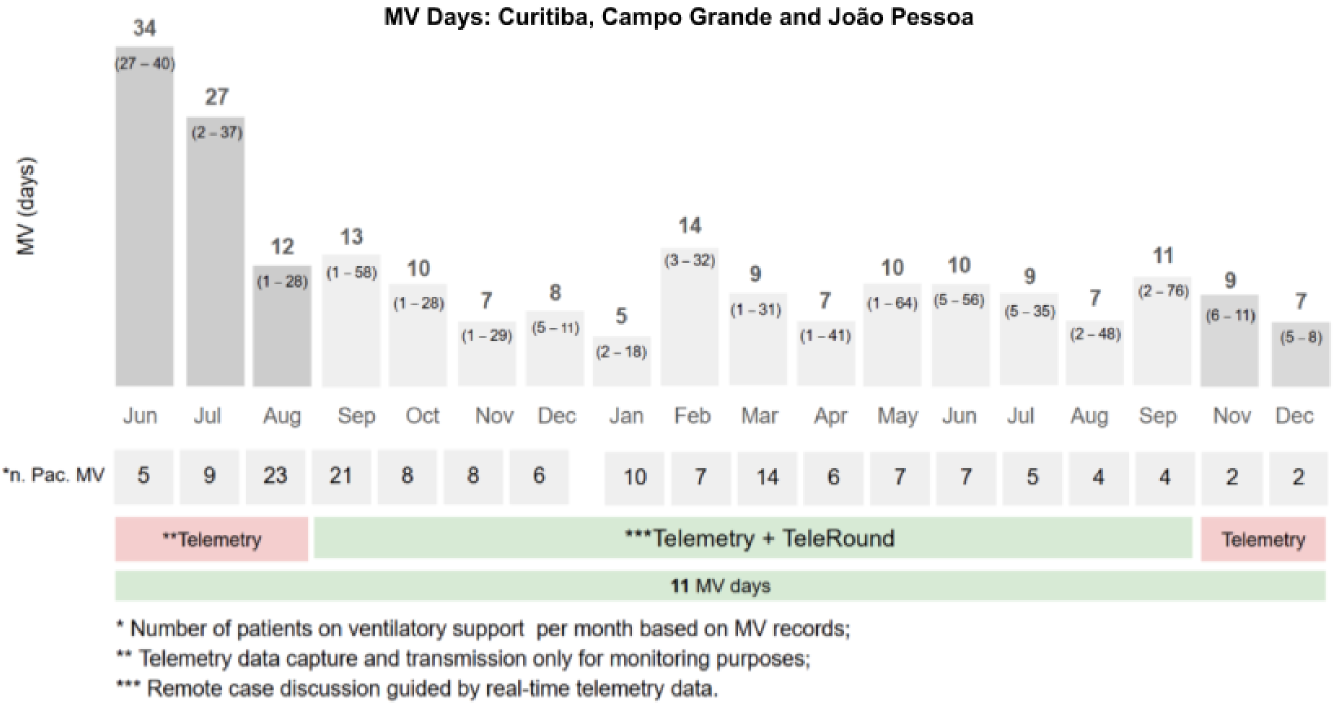

MV - mechanical ventilation.

Figure 6S - Days on ventilator support

TELE-ROUND AND PHYSIOLOGICAL DETAILING

Baseline characteristics of patients' peripheral oxygen saturation/fraction of inspired oxygen - hypoxemic respiratory failure

One-way continuous telemetry data is used is in the assessment and adjustment of procedures recorded by INTEGRARE® during tele-rounds, which generate valuable information on the disease and the approaches adopted by multidisciplinary ICU teams. For example, retrospectively, using the INTEGRARE® historical tool, it was possible to automatically characterize the Hypoxemic Respiratory Failure profile by combining MM and MV data. In this example, in the absence of blood gas data, data from the mechanical ventilator and the MM were used to characterize the severity of patients on mechanical ventilation, replacing the arterial partial pressure of oxygen/fraction of inspired oxygen (PaO_2/FiO_2) ratio with the peripheral oxygen saturation/fraction of inspired oxygen (SpO_2/FiO_2) ratio (S/F).

According to tables 1S and 2S, the 148 patients who were on mechanical ventilation during the proof-of-concept were characterized along three axes: S/F level, ventilatory parameters, and ICU stay time interval. First, they were grouped into four levels (all patients, mild, moderate, and severe) according to each patient's median S/F. Second, they were cross-sectionally correlated with the median ventilatory parameters. Third, they were analyzed over two time periods: the first 6 hours of ICU stay and 60 - 72 hours of ICU stay.

In this example, the intention was to provide a hypoxemic respiratory failure profile of the patient population, including severity level, respective ventilatory management during ICU admission, and evolution after three days. However, the same set of tools allows for detailed characterization at any time interval or physiological segmentation expressed by MM and/or MV.

Table 2S - Baseline characteristics of patients' peripheral oxygen saturation/fraction of inspired oxygen - hypoxemic respiratory failure - first 6 hours

SpO_2/FiO_2	All (100%)	Mild (37,93%)	Moderate (49,13%)	Severe (12,93%)
FiO_2 (95%CI)	45.0 (21 - 100)	30.0 (21 - 40)	50.0 (40 - 60)	100.0 (65 - 100)
$SpO_2 \leq 94\%$	92.0 (87 - 94)	93.0 (92 - 94)	91.0 (88-93)	91.0 (87 - 93.5)
$SpO_2 > 94\%$	97.0 (95 -100)	97.5 (95 - 99)	97.0 (95 - 100)	97.0 (96 - 99)
Set respiratory rate, mean (95%CI)	20.0 (6 - 38)	20.0 (6 - 34)	19.5 (6 - 34)	20.0 (6 - 38)
Total respiratory rate, mean (95%CI)	31.3 (10 -131.5)	27.8 (11 - 68.8)	40.8 (11 - 131.5)	33.2 (15 - 69.3)
VT (95%CI), mL/kg PBW (sec)	390.0 (71 - 803)	419.0 (311 - 798)	384.0 (97.5 - 803)	327.0 (71 - 643.5)
Control ventilation mode/assist-control	213h51	108h37	73h49	56h4
Spontaneous ventilation mode	56h22	35h25	18h28	2h28
Set PEEP (cmH_2O) (95% CI)	8.0 (4 - 16)	7.0 (5 - 16)	8.0 (4 - 15)	9.0 (5 - 10)
Peak pressure (cmH_2O), mean (95%CI)	20.7 (7.7 - 38.4)	20.0 (11.6 - 38.4)	22.0 (7.7 - 36.1)	22.5 (10.6 - 35.6)
Standardized minute ventilation	8.8 (3.5 -17.4)	8.7 (5.0 - 13.3)	9.1 (3.6 - 17.6)	8.0 (4.8 - 11.2)

SpO_2 - peripheral oxygen saturation; FiO_2 - fraction of inspired oxygen; VT - tidal volume; PBW - predicted body weight; PEEP - positive end-expiratory pressure.

Table 3S - Baseline characteristics of patients' peripheral oxygen saturation/fraction of inspired oxygen - hypoxemic respiratory failure - 60 hours - 72 hours

SpO_2/FiO_2	All (100%)	Mild (33,3%)	Moderate (27%)	Severe (16%)
FiO_2 (95% CI)	45.0 (30 - 100)	40.0 (30 - 40)	45.0 (40 - 60)	97.5 (70 - 100)
$SpO_2 \leq 94\%$	93.0 (60 - 94)	93.0 (85 - 98)	93.5 (93 - 94)	92.5 (60 - 94)
$SpO_2 > 94\%$	96.0 (95 - 100)	96 (95 - 98)	96 (95 -100)	97 (95 - 99)
Set respiratory rate, mean (95%CI)	20.0 (6 - 38)	23.0 (6 - 38)	20.0 (6 - 34)	19.5 (14 - 28)
Total respiratory rate, mean (95%CI)	31.05 (12 - 80.9)	33.2 (12 - 67.2)	28.4 (16 - 68)	47.2 (22.5 - 80.9)
VT (mL/kg) PBW sec (95% CI)	358.2 (218 - 701)	360 (218 - 701)	342.0 (256 - 519)	359.0 (271 - 435)
Control ventilation mode/assist-control	420h4	228h31	178h4	71h31
Spontaneous ventilation mode	69h34	44h2	35h52	13h40
Set PEEP (cmH_2O) (95% CI)	8.0 (5 - 20)	8.0 (5 - 20)	8.0 (5 - 16)	10.0 (7 - 18)
Peak pressure (cmH_2O), mean (95%CI) cmH_2O	22.3 (12 - 37)	20.5 (12 - 35.5)	22.8 (12.7 - 32.6)	30.0 (21.2 - 37)
Standardized minute ventilation	8.2 (5 - 15.1)	8.4 (5.7 - 11)	8.7 (5 - 12)	7.7 (6.6 - 15)

SpO_2 - peripheral oxygen saturation; FiO_2 - fraction of inspired oxygen; VT - tidal volume; PBW - predicted body weight ; PEEP - positive end-expiratory pressure.

Characterization of ventilatory asynchrony (reverse triggering), freezing, and measuring the ventilatory curve

The WEB INTEGRARE® graphical tools help the ICU multidisciplinary team and the remote medical specialist to analyze the patient's physiological events by combining numerical data and physiological waves and, simultaneously, discuss these data via video call. In a tele-round context, for example, when it was important to know if mechanical ventilation was protective, the remote intensive care specialist would ask the local ICU team to perform an inspiratory pause directly on the mechanical ventilator. Simultaneously, visualizing the ventilatory curves through the WEB INTEGRARE® interface, the remote specialist would activate the ventilation curve freeze mechanism and, consequently, obtain the plateau pressure and the driving pressure. Thus, it makes it possible to explain, with a real example, that ventilation was protective. If the ventilation was not protective, it was also possible to show the multidisciplinary team and teach strategies to achieve this goal.

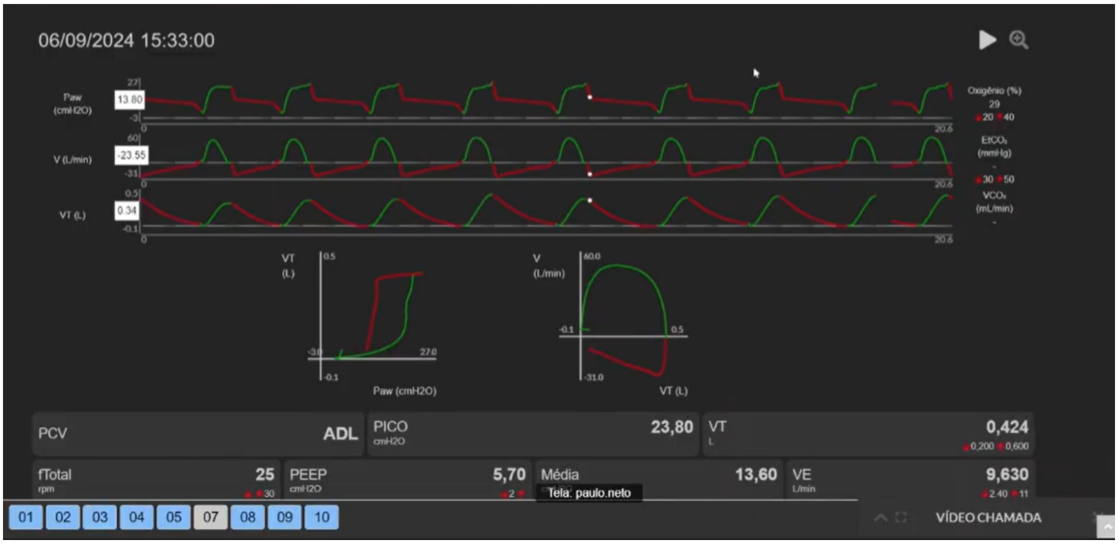

Figure 7S - WEB INTEGRARE® module - freezing and measuring ventilatory curve.

Another very common example was the identification of patient-ventilator asynchronies, where the remote specialist would identify and freeze the asynchrony via the INTEGRARE® web interface, explaining what the asynchrony was and how it should be corrected. It was possible to visualize the correction and the disappearance of the asynchrony in real time, benefiting the patient.

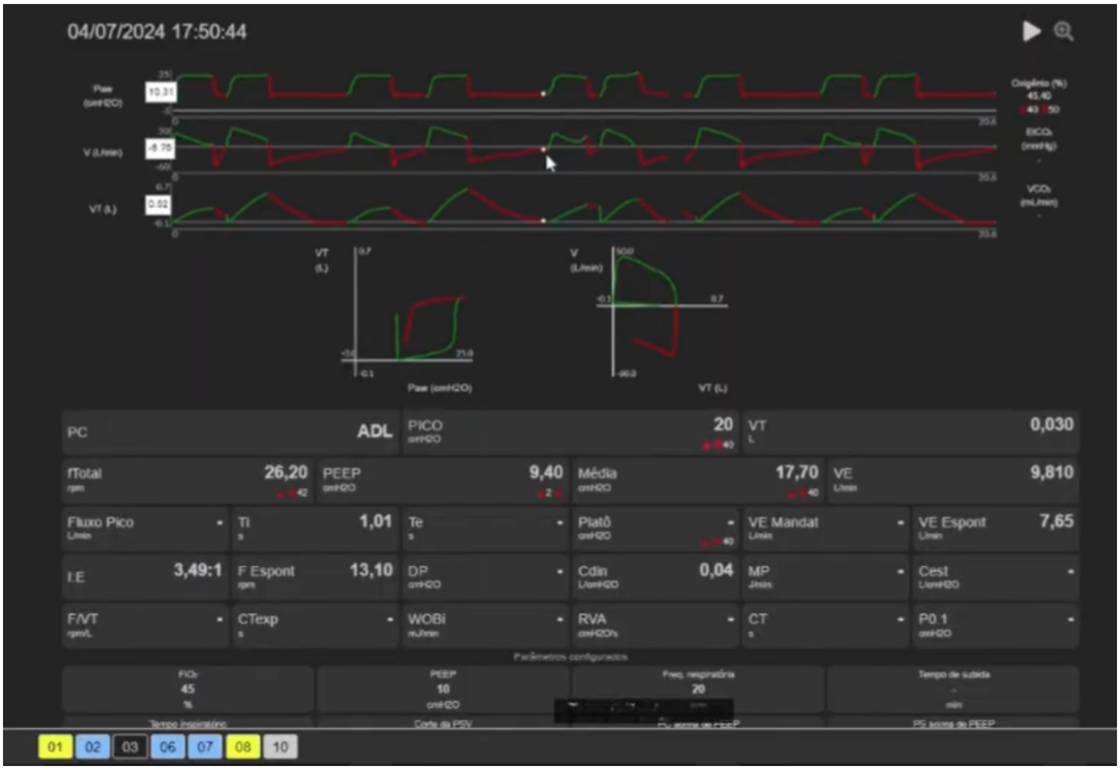

Figure 8S - WEB INTEGRARE® module - characterization of ventilatory asynchrony (reverse triggering), freezing and measuring ventilatory curve.
